# Comparison of Eustachian tube ventilation function between cleft palate and normal patients using sonotubometry

**DOI:** 10.1016/j.jpra.2021.04.003

**Published:** 2021-04-24

**Authors:** Dini Widiarni Widodo, Adila Hisyam, Widayat Alviandi, Muchtaruddin Mansyur

**Affiliations:** a Department of Otolaryngology, Cipto Mangunkusumo Hospital – Universitas Indonesia, P.Diponegoro Street no. 71, Senen, Central Jakarta, 10430, Indonesia; b Department of Public Health and Community, Cipto Mangunkusumo Hospital – Universitas Indonesia, P.Diponegoro Street no. 71, Senen, Central Jakarta, 10430, Indonesia

**Keywords:** Eustachian tube function, Cleft palate, Sonotubometry

## Abstract

**Objective:**

To compare Eustachian tube ventilation function between cleft palate subjects and normal subjects using sonotubometry.

**Method:**

A comparative cross-sectional study was conducted at the Department Otolaryngology-Head and Neck Surgery of Ciptomangunkusumo National Hospital, Universitas Indonesia, Jakarta, from June 2013 to January 2014.There were 31 subjects with cleft palate and 62 healthy subjects aged ≤18 years, and both groups were matched according to age. Each subject underwent ear, nose, and throat examination with Veau classification and sonotubometry, a new assembly test in Indonesia. The results of the sonotubogram (the number of Eustachian tube openings, amplitude enhancement in dB, and the duration of Eustachian tube opening in ms) were then analyzed with SPSS using chi-square and Mann–Whitney tests.

**Results:**

Subjects with cleft palate had lower Eustachian tube function than healthy subjects using three sonotubometry parameters (*p* < 0.001). The proportion of Eustachian tube dysfunction based on the Veau classification was significant (*p* < 0.001). In multivariate analysis, several determinant factors of Eustachian tube dysfunction were found, such as adenoid hypertrophy (risk factor6.46), the number of Eustachian tube openings (risk factor 36.21), and higher Veau classification (risk factor 10.41).

**Conclusion:**

Sonotubometry could be used to assess parameters of Eustachian tube function. Subjects with cleft palate have a higher risk of having Eustachian tube dysfunction, as do subjects with adenoid hypertrophy.

## Introduction

Eustachian tubes have three physiological functions: ventilation (balancing air pressure with atmospheric pressure), drainage, and clearance of the middle ear secretion products into the nasopharynx. The Eustachian tube also protects the middle ear from sound pressure and controls nasopharyngeal secretions. The function works when the Eustachian tube actively and intermittently opens, resulting in contraction of the tensor veli palatine muscle during swallowing.^1^

Subjects with cleft palate have several problems, including dysfunction of the Eustachian tube.^2^ This dysfunction is caused by abnormal insertion and hypoplasia of the levator veli palatine and tensor veli palatine muscles. Leuwer et al. found an abnormal insertion of the tensor veli palatine muscle in the lateral wall of the chondroid part of the Eustachian tube in subjects with cleft palate.^3^ This abnormal insertion causes obstruction of the Eustachian tube and results in a high incidence of middle ear infection. The Eustachian tube turns towards the caudal instead of running straight from the posterolateral to the anteromedial, changing the muscle's pathway and insertion into the palate. Therefore, the functions of tensor and levator veli palatine muscles are ineffective in the opening of the Eustachian tube in subjects with cleft palate.^4^

Various tests have been developed to assess Eustachian tube function to diagnose and identify ET dysfunction. Sonotubometry can determine whether there is an ET opening during swallowing or other maneuvers to rule out some of the causes of Eustachian tube dysfunction.^5^ This device allows examination under physiological conditions with the principle of sound being delivered to the Eustachian tube in the nasopharynx to the middle ear.

According to Martino et al., normal subjects will have ≥5 Eustachian tube openings and 2.8 ms–7.2 s (median 459 ms, mean 705.4 ms, DS ±711.3 ms) opening durations during a 10-s maneuver. Normal amplitude enhancement is ≥5 dB.^6^

Several studies on the function of the Eustachian tube in subjects with cleft palate have been conducted, but there have been only a few studies using sonotubometry to assess Eustachian tube function in Indonesia.^7^^,^^8^ This study aimed to compare Eustachian tube function using sonotubometry between subjects with cleft palates and normal Indonesian subjects.

## Methods

This comparative cross-sectional study involved 31 subjects with cleft palate and62 normal subjects under 18 years of age. The inclusion criteria were subjects with unrepaired cleft palate without acute allergic rhinitis and acute rhinosinusitis. Normal subjects must have normal tympanometry (type A), normal adenoid glands, and be free from ear and nose infections. Both subject groups were between the ages of 0–18 years old and were paired (matched). All subjects were examined using nasoendoscopy and tympanometry to match the inclusion criteria. The type of cleft palate was determined with direct visualization using Veau classification.

**Figure 1:**
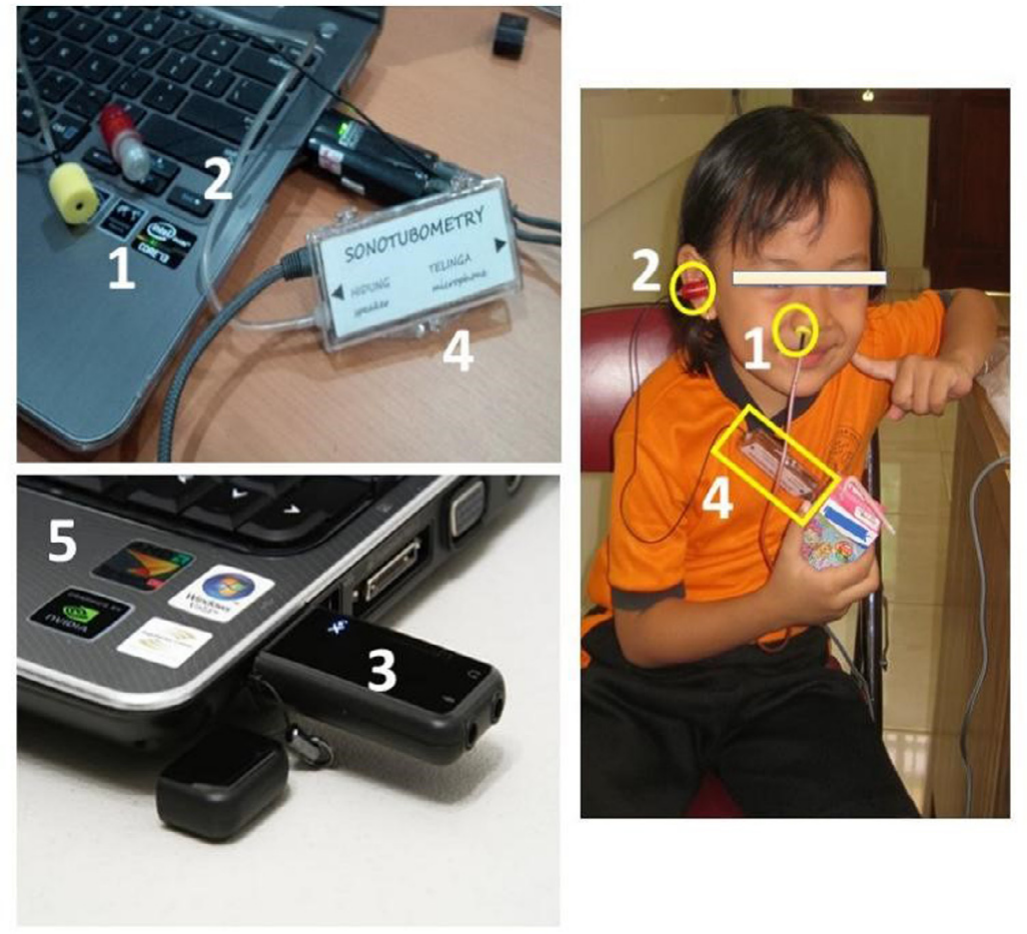
Sonotubometry device: 1. Hearing Aid speaker (ear tone 3A); 2. Type CM 120 condenser microphone; 3. SB1140 creative sound card; 4. SN 002020; 5. Laptop.

The sonotubometry device (Figure 1) was made from a Hearing Aid speaker (ear tone 3A), a type CM 120 condenser microphone, an SB1140 creative sound card, Adobe Audition CS6 software, and a USB soundcard (creative sound blaster). The calibration tool used a sound level meter CS 20 type, SN 002020. A coefficient of variance (CoV) ≤ 0.10 was calculated to determine the validity of sonotubometry. The research parameter results were as follows: the number of Eustachian tube openings was 0.1, the amplitude was enhanced by 0.09, and the duration of Eustachian tube opening was 0.08.

The researcher used a custom-made sonotubometry device that had been previously validated. A constant sound was inserted into the nasal cavity, and a microphone was placed in the outer ear canal to record changes in sound intensity through the Eustachian tubes and the middle ear. Subjects were tested in a quiet room in a sitting or lying position. The head position must be higher from the body. The speaker probe was inserted into the anterior nares, and the microphone probe was inserted into the ipsilateral outer ear canal. A sound of 8000 Hz pure tone was played to avoid false-negative results through the speaker with an intensity of 60 dBHL (equivalent to 10 dBFS) recorded in the Adobe Audition CS6 program. The sound was captured by the microphone and recorded as a graph on the computer. Subjects were instructed to swallow drinking water quickly (10 swallows within 10 s). Children had to be cooperative to perform the maneuver. Uncooperative children were excluded as subjects. The test was repeated twice with the same device.

The sonotubogram results were the number of Eustachian tube openings in times, an increase in amplitude in dB, and the duration of Eustachian tube opening in ms (millisecond).

The validation of the method was performed in a healthy subject who was tested with sonotubometry 3 times in each ear. Assessment of the three parameters and statistics was performed using the coefficient of variance (CoV), which was declared valid if ≤0.10. The CoV results for the number of Eustachian tube openings were 0.1, an increase in amplitude of 0.09, and the duration of the Eustachian tube opening was 0.08.

Statistical analysis was performed using SPSS 20.0 software. Chi-square tests and Mann-Whitney U tests were used to compare the groups. Multivariate analysis was performed to determine the correlations between groups.

## Results

In this study, there were 93 subjects aged ≤18 years consisting of 31 subjects with cleft palate and 62 normal subjects, who were then matched according to age. The research lasted for eight months, from June 2013 to January 2014. There were 24 subjects with cleft palate aged ≤7 years old (77.4%), dominated by 19 male subjects (61.3%). The number of cases of adenoid hypertrophy in the subjects with cleft palate was 22 (71.0%), with a low risk of atopy history in 27 (87.1%)(Table 1).

**Table 1:** Characteristics of subjects.

Variable		Cleft palate (*n* = 31)		Healthy subject(*n* = 62)	
		*n*	(%)	*n*	(%)
Age	≤7 years old	24	(77.4)	48	(77.4)
	>7–18 years old	7	(22.6)	14	(22.6)
Sex	Male	19	(61.3)	23	(37.1)
	Female	12	(38.7)	39	(62.9)
Adenoid hypertrophy	None	9	(29)	40	(64.5)
	Hypertrophy	22	(71)	22	(35.5)
Low risk		27	(87.1)	40	(64.5)

Most of the subjects with cleft palate were in Veau II classifications, 20 (64.5%). The least common group was those with Veau IV classification, 2 subjects (6.5%) with complete bilateral palate clefts. In collecting data, not all subjects were able to be examined in either ear due to massive serum prop. We collected twelve subjects with cleft palate who underwent unilateral ear examination; nineteen subjects with cleft palate and 62 normal subjects underwent bilateral ear examination. Data were analyzed based on the total ears examined (*n* = 174).

**Figure 2:**
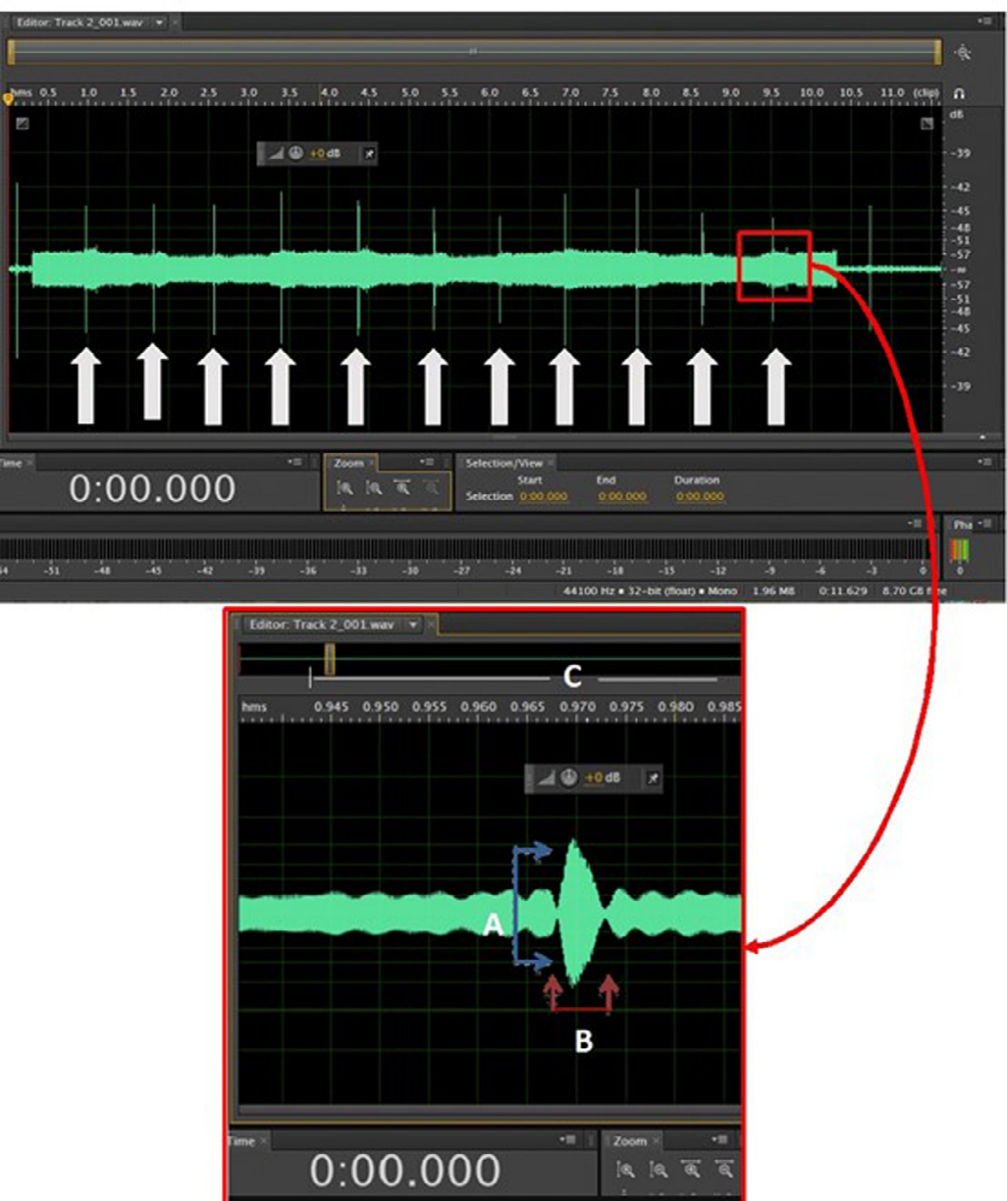
Sonotubogram and the parameters. Thick white arrows show the time of Eustachian tube opening within 10 s. The blue arrow (A) shows amplitude enhancement in decibels (dB). Red arrows (B) show the duration of Eustachian tube opening in milliseconds (ms). The opening duration is measured with a ruler above the wave (C). (For interpretation of the references to color in this figure legend, the reader is referred to the web version of this article.)

The sonotubogram shows the opening of the Eustachian tube within 10 s, illustrated by an arrow. In addition, an increase in sonotubogram amplitude can be seen to assess the opening of the Eustachian tube and the opening duration of the Eustachian tube (Figure 2).

The fifty ears of subjects with cleft palate had a median Eustachian tube function opening 5 (interquartile range: 4) times lower than that of normal subjects (7.5 times). Amplitude enhancement of the cleft palate was obtained with a median value of 5.45 dB (interquartile range: 3.58 dB), which was lower than that in normal subjects (9.2 dB, (interquartile range: 3.62 dB)). The median value of the opening duration of the Eustachian tube in the cleft palate was 155 ms (interquartile range: 103 ms), which was longer than that in normal subjects (96.85 dB (interquartile range: 25.25 dB)). All three parameters obtained a *p*-value < 0.001, which was statistically significant (Table 2).

**Table 2:** Comparison of Eustachian tube opening number, amplitude enhancement in dB and Eustachian tube opening duration in ms to cleft palate and normal subjects in each ear (*n* indicates number of ears).

	Cleft palate (*n* = 50) median (interquartile range)	Healthy subject (*n* = 124) median (IQR)	*p*
Opening number	5 (4)	7.5 (4)	0.000*
Amplitude enhancement (dB)	5.45 (3.58)	9.2 (3.62)	0.000*
Opening duration (milliseconds)	155 (103)	96.85 (25.25)	0.000*

⁎ using the Mann–Whitney test.

Comparison of Eustachian tube function proportions in the study subjects in each ear showed statistically significant results (*p* < 0.001) using the chi-square test. Cases with Veau III and IV cleft palate classification revealed Eustachian tube ventilation impairment in 5 out of 10 ears compared with the Eustachian tube dysfunction that occurred in Veau I and II cases, with 20 out of 40 ears (50%), and in healthy subjects, with as many as 3 of 124 ears (2.4%) (Table 3).

**Table 3:** Comparison of Eustachian tube function abnormality proportion based on the classification of cleft palate and healthy subjects each ear (*n* indicates the number of ears).

		Eustachian tube ventilation function (*n* = 174)		*p*
		Abnormal *n* (%)	Normal *n* (%)	
Cleft palates	Veau III and IV	5 (50%)	5 (50%)	0.000*
	Veau I and II	20 (50%)	20 (50%)	
Healthy subjects		3 (2.4%)	121 (97.6%)	

⁎ Using the chi-square test.

Multivariate analysis was performed to assess factors related to Eustachian tube dysfunction. The results obtained showed statistically significant adenoid hypertrophy with *p* = 0.006 (*p* < 0.01) for Eustachian tube function. Adenoid hypertrophy had a possible increased risk for impaired Eustachian tube function (6.46-fold with a 95% CI (1.69–24.75)). There was a correlation between the number of Eustachian tube openings and the cleft palate of Veau III and IV classifications.

The number of Eustachian tube openings (<5 times) had 1536.21-fold increased risk of Eustachian tube ventilation dysfunction with a 95% CI (9.10–143.99). The proportion of Eustachian tube ventilation dysfunction in the subjects with Veau III and IV palate cleft risk was 10.41-fold increased, with a 95% CI (1.18–91.82) (Table 4).

**Table 4:** Multivariate analysis for adenoid hypertrophy, Eustachian tube opening number <5x and subjects with cleft palate classification of Veau III and IV to Eustachian tube ventilation function in each ear.

Variable	Adjusted OR (CI 95%)	*p*
Adenoid hypertrophy	6.46 (1.69–24.75)	0.006
Eustachian tube opening number <5x	36.21 (9.10–143.99)	0.000
Subject with cleft palate classifications of Veau III and IV	10.41 (1.18–91.82)	0.035

## Discussion

Most of the subjects with cleft palates were male. According to Noorollahian et al., the prevalence of oral clefts tends to be higher in male than in female patients. There was no scientific explanation related to the difference between cleft palate and sex.^9^

Subjects with cleft palate were found mostly to have adenoid hypertrophy due to greater infection and reflux factors to the nasopharynx than healthy subjects. Khayat et al. stated that adenoids will develop to the largest size at the age of 7 years because at younger ages, they often cause complaints associated with the small nasopharyngeal space and an increase in the frequency of upper respiratory tract infections.^10^ Adenoid hypertrophy had a 6.46-fold increased possibility of becoming Eustachian tube ventilation dysfunction. This was in accordance with Cassano, quoted by Acharya et al., who stated that the increase in the degree of adenoid hypertrophy correlated with the onset of effusion otitis media, which was statistically significant (*p*-value 0.0002). Grade 4 adenoid hypertrophy alone had a significant relationship with the onset of OME. Adenoid hypertrophy was also related to speech function.^11^ In Yassi et al.’s study related to adenoid hypertrophy with the cleft palate, differences in nasal scores were obtained, which illustrated the outcome of subjects with cleft palate and normal subjects. Adenoid hypertrophy was significantly associated with nasalance scores in subjects with cleft palate.^8^^,^^12^

In this study, the Veau classification was used because based on Shah et al., which is related to the morphological classification of cleft palate, the Veau classification has been widely used because it is quite detailed, clear, and covers all types of cleft lip and palate.^13^

The difference from this study was that in those studies, the research subjects were of adult age. This implied subject compliance while performing the sonotubometry testing. In our study, which used children under 18 years old as subjects, bias can occur in setting the time limit of the swallowing process.

In a sonotubometry examination, several provocations were made to open the Eustachian tube, such as swallowing water, swallowing without water, Valsalva, and yawning. Each maneuver was carried out 4 times in a row. Other maneuvers were carried out before and after the administration of decongestants with 0.1% oxylomazoline drops. The results of various maneuvers show a variety of tubal activities. The highest incidence of Eustachian tube opening during swallowing was 57% (28.8% swallowing without water and 28.2% swallowing with water). The duration for all tubal openings was between 2.8 ms and 7.2 s (median 459 ms, mean 705.4 ms, SD ± 711.3 ms). The median increased sound intensity at Eustachian tube opening was 14.0 dB (0.5–40 dB, average 15.2 dB, SD 7.8 dB). Topical decongestants with xylometazoline made the sonotubogram curve more pronounced and showed no significant difference between the two treatments (*p* < 0.89 NS, *p* < 0.31 NS).

The median value of the number of Eustachian tube openings in the subjects with cleft palate was 5 times, which was lower than that in normal subjects (7.5 times). Thus, both could be considered to have a normal Eustachian tube opening function. However, in this examination, there was quite high subjectivity related to the characteristics of the subject. The Avoort et al. study resembled this study, in which the subjects were 5- to 9-year-old cleft palate children, and the only parameter measured was the number of Eustachian tube openings. In the other study, the comparison of Eustachian tube function in the cleft palate was 56%, while in healthy children, it was 89%. Additionally, in that other study, it was found that the number of Eustachian tubes opening was less than 5.^1^ Martin et al. stated that during the swallowing process, the tensor veli palatine muscle plays a role in opening the Eustachian tube ostium.^14^ The mechanism of Eustachian tube dysfunction in the cleft palate is due to the abnormality of the cranium base and insertion of the tensor veli palatini muscles, which causes constriction of the Eustachian tube ostium.

The proportion of amplitude enhancement in the subjects with cleft palate obtained a smaller median value of 5.45 dB compared with that of normal subjects, 9.2 dB. In this parameter, it was said that the Eustachian tube function was normal when the sound intensity increased ≥ 5 dB. The proportion of Eustachian tube dysfunction based on amplitude enhancement of less than 5 dB was obtained at 100%. The median values of amplitude enhancement in the studies by Martino et al. and Kitajima et al. in healthy adult subjects were slightly higher than in this study, while the median value of amplitude enhancement in subjects with cleft palate was lower but still within normal limits.^6^^,^^15^ This examination was more objective because the assessment was based on the sound that was caught by the microphone in the external acoustic canal and was not influenced by the subject. One of the most frequent types of Eustachian tube dysfunction is when the lumen of the cartilage fails to open during swallowing activity.^16^ Therefore, the sound entering from the anterior nares cannot or only slightly enters through the Eustachian tube ostium during the process of swallowing so that the sound cannot be captured by the microphone in the ear canal.

The duration of Eustachian tube opening in the subjects with cleft palate was lengthened by 155 ms, while the duration of Eustachian tube opening in normal subjects was 96.85 ms shorter. Martino et al., in his research, obtained the value of Eustachian tube opening at 355.6 ms (SD ± 335.1, median 265.0 ms), while in Kitajima et al., the normal average duration of Eustachian tube opening was 422.5 ± 178.2 ms. The researchers found a normal number for the duration of Eustachian tube opening with a range of 66.1–778.9 ms.^6^^,^^15^ Meanwhile, when compared with the healthy subjects in this study, the duration of opening in the subjects with cleft palate was longer. However, when relaxation occurs, the muscles slow down to their original position, and the duration of Eustachian tube opening is extended. This difference is probably due to anisometric paratubal muscles.

In addition, in this study, 50% normal Eustachian tube function was found in the patients with cleft palate. This is consistent with Leuwer's opinion showing that the physiological function of the Eustachian tube depends on certain signs that can change the direction of muscle tension, called hypomoclia. There are three hypomoclia that have been identified: pterygoid hamulus, Ostmann's fatty tissue between the Eustachian tube lumen and palatini tensor veli muscle, and medial pterygoid muscle. Leuwer et al. found that all subjects without pathological abnormalities of the ear had an intact pterygoid hamulus and intact tensor veli palatini muscle.^6^ In this study, 7 subjects with cleft palate were found to have normal Eustachian tube function. In subjects with unoperated cleft palate, there is a thin but still complete tensor muscle and an intact pterygoid hamule.

The development of the Eustachian tube is influenced by the growth and development of the middle part of the face, especially in the more extensive cases of cleft palate and lips. According to Bluestone et al., there was no anatomical obstruction of the Eustachian tube in subjects with cleft palate, but there was a failure in the opening mechanism of the Eustachian tube as an underlying defect (functional obstruction as opposed to anatomy).^16^ The discovery of other anatomical abnormalities, such as cartilage and abnormal lumen, the insertion ratio of the m. tensor veli palatini into the cartilage, reduced attachment of tensor veli palatini muscles into the lateral lamina cartilage and elastin deficiency in the cartilage region, most likely are the causes of the functional obstruction.

A multidisciplinary team is needed to manage the various impacts from cleft palate. Reconstructive surgery in conjunction with various other medical fields, such as otolaryngologists, orthodontists, and audiologists, is needed. Surgical techniques for cleft lip and palate continue to develop, and many techniques currently exist to repair cleft palate.^17^ The optimum age for palatoplasty surgery is 6–12 months. Many other centers perform palatoplasty between the ages of 12–18 months, while very few perform this surgery in individuals aged 10–12 years. Ideally, the development of speech (babbling) is one of the indicators for the reconstruction of the palate. The principle of palatoplasty is to close the defect, correct the position of the abnormal soft palate muscles, especially the levator veli palatini, reconstruct muscle ties, and retroposition the soft palate as much as possible so that during speaking, the posterior part of the soft palate approaches the posterior pharyngeal wall, minimizing or eliminating space on the side of the palate on the nasal or oral surface, with tension-free stitching, closure of the hard palate with two layers, and closure on the soft palate with 3 layers.^18^ Smith et al. stated that dysfunction of the Eustachian tube heals faster with double Z-plasty.^19^

## Conclusion

Subjects with cleft palate have significantly lower numbers of Eustachian tube openings, lower amplitude enhancement, and longer Eustachian tube openings than normal subjects. The classification of Veau III and IV cleft palate is characterized by Eustachian tube ventilation impairment in 5 out of 10 ears compared with Eustachian tube dysfunction that occurs in Veau I and II, comprising 20 out of 40 ears. Adenoid hypertrophy was a possible risk for impaired Eustachian tube function (6.46 times, with a 95% CI (1.69–24.75)). The lower number of Eustachian tube openings (<5 times) had a 1536.21-fold increased risk of Eustachian tube ventilation dysfunction, and subjects with Veau III and IV cleft palate classification had a 10.41-fold higher risk of ventilation dysfunction.

It is recommended to perform sonotubometry examinations before and after surgery to determine Eustachian tube functional improvement.

## Declaration of Competing Interest

The authors declare that they have no known competing financial interests or personal relationships that could have appeared to influence the work reported in this paper.
